# Supplementary material for: Iterative improvement in the automatic modular design of robot swarms
Source: PeerJ Comput Sci. 2020 Dec 7;6:e322. doi: 10.7717/peerj-cs.322 (PMC7924708; doi:10.7717/peerj-cs.322)
Supplement: Supplemental Information 3 [file peerj-cs-06-322-s003.zip › argos3/doc/api/standalone/a00609_source.html]

ARGoS: plugins/simulator/physics\_engines/dynamics2d/dynamics2d\_gripping.h Source File


- Main Page
- Related Pages
- Namespaces
- Classes
- Files

- File List
- File Members

# plugins/simulator/physics\_engines/dynamics2d/dynamics2d\_gripping.h

Go to the documentation of this file.

```
00001 
00007 #ifndef DYNAMICS2D_GRIPPING_H
00008 #define DYNAMICS2D_GRIPPING_H
00009 
00010 namespace argos {
00011    class CDynamics2DEngine;
00012    class CDynamics2DGripper;
00013    class CDynamics2DGrippable;
00014    class CGripperEquippedEntity;
00015 }
00016 
00017 #include <argos3/plugins/simulator/physics_engines/dynamics2d/chipmunk-physics/include/chipmunk.h>
00018 #include <argos3/plugins/simulator/entities/gripper_equipped_entity.h>
00019 #include <list>
00020 
00021 namespace argos {
00022 
00023    /****************************************/
00024    /****************************************/
00025 
00026    class CDynamics2DGripper {
00027 
00028    public:
00029 
00030       typedef std::list<CDynamics2DGripper*> TList;
00031 
00032    public:
00033 
00034       CDynamics2DGripper(CDynamics2DEngine& c_engine,
00035                          CGripperEquippedEntity& c_gripper_entity,
00036                          cpShape* pt_gripper_shape);
00037 
00038       ~CDynamics2DGripper();
00039 
00040       inline bool IsGripping() const {
00041          return m_pcGrippee != NULL;
00042       }
00043 
00044       inline bool IsLocked() const {
00045          return m_cGripperEntity.IsLocked();
00046       }
00047 
00048       inline CGripperEquippedEntity& GetGripperEntity() {
00049          return m_cGripperEntity;
00050       }
00051 
00052       inline cpShape* GripperShape() {
00053          return m_ptGripperShape;
00054       }
00055       
00056       inline CDynamics2DGrippable* GetGrippee() {
00057          return m_pcGrippee;
00058       }
00059 
00060       inline cpConstraint* GetConstraint() {
00061          return m_tConstraint;
00062       }
00063 
00064       void CalculateAnchor(cpArbiter* pt_arb);
00065 
00066       void Grip(CDynamics2DGrippable* pc_grippee);
00067 
00068       void Release();
00069 
00070    private:
00071 
00072       CDynamics2DEngine&      m_cEngine;
00073       CGripperEquippedEntity& m_cGripperEntity;
00074       cpShape*                m_ptGripperShape;
00075       CDynamics2DGrippable*   m_pcGrippee;
00076       cpVect                  m_tAnchor;
00077       cpConstraint*           m_tConstraint;
00078 
00079    };
00080 
00081    /****************************************/
00082    /****************************************/
00083 
00084    class CDynamics2DGrippable {
00085 
00086    public:
00087 
00088       CDynamics2DGrippable(CEmbodiedEntity& c_entity,
00089                            cpShape* pt_shape);
00090 
00091       ~CDynamics2DGrippable();
00092 
00093       inline CEmbodiedEntity& GetEmbodiedEntity() {
00094          return m_cEmbodiedEntity;
00095       }
00096 
00097       inline cpShape* GetShape() {
00098          return m_ptShape;
00099       }
00100 
00101       inline bool HasGrippers() const {
00102          return !m_listGrippers.empty();
00103       }
00104 
00105       void Attach(CDynamics2DGripper& c_gripper);
00106 
00107       void Remove(CDynamics2DGripper& c_gripper);
00108 
00109       void Release(CDynamics2DGripper& c_gripper);
00110 
00111       void ReleaseAll();
00112 
00113    private:
00114 
00115       CEmbodiedEntity&          m_cEmbodiedEntity;
00116       cpShape*                  m_ptShape;
00117       CDynamics2DGripper::TList m_listGrippers;
00118 
00119    };
00120 
00121    /****************************************/
00122    /****************************************/
00123 
00124    extern int BeginCollisionBetweenGripperAndGrippable(cpArbiter* pt_arb,
00125                                                        cpSpace* pt_space,
00126                                                        void* p_data);
00127 
00128    extern int ManageCollisionBetweenGripperAndGrippable(cpArbiter* pt_arb,
00129                                                         cpSpace* pt_space,
00130                                                         void* p_data);
00131    
00132    extern void AddConstraintBetweenGripperAndGrippable(cpSpace* pt_space,
00133                                                        void* p_obj,
00134                                                        void* p_data);
00135    
00136    extern void RemoveConstraintBetweenGripperAndGrippable(cpSpace* pt_space,
00137                                                           void* p_obj,
00138                                                           void* p_data);
00139    
00140    /****************************************/
00141    /****************************************/
00142 
00143 }
00144 
00145 #endif
```

---

Generated on 10 Jul 2018 for ARGoS by 
 1.6.1
